# Smoking may be more harmful to vasospastic angina patients who take antiplatelet agents due to the interaction: Results of Korean prospective multi-center cohort

**DOI:** 10.1371/journal.pone.0248386

**Published:** 2021-04-02

**Authors:** Seong-Sik Cho, Sang-Ho Jo, Hyun-Jin Kim, Min-Ho Lee, Won-Woo Seo, Hack-Lyoung Kim, Kwan Yong Lee, Tae-Hyun Yang, Sung-Ho Her, Seung Hwan Han, Byoung-Kwon Lee, Keun-Ho Park, Seung-Woon Rha, Hyeon-Cheol Gwon, Dong-Ju Choi, Sang Hong Baek

**Affiliations:** 1 Department of Occupational and Environmental Medicine, College of Medicine Dong-A University, Busan, Korea; 2 Department of Preventive Medicine, College of Medicine Dong-A University, Busan, Korea; 3 Cardiovascular Center, Hallym University Sacred Heart Hospital, Anyang-si, South Korea; 4 Division of Cardiology, Department of Internal Medicine, Hanyang University College of Medicine, Seoul, South Korea; 5 Division of Cardiology, Department of Internal Medicine, Soonchunhyang University Seoul Hospital, Seoul, South Korea; 6 Division of Cardiology, Department of Internal Medicine, Kangdong Sacred Heart Hospital, Hallym University College of Medicine, Seoul, South Korea; 7 Division of Cardiology, Department of Internal Medicine, Boramae Medical Center, Seoul National University College of Medicine, Seoul, Korea; 8 Department of Cardiovascular Medicine, Incheon St. Mary’s Hospital, The Catholic University of Korea, Incheon, South Korea; 9 Department of Cardiovascular Medicine, Busan Paik Hospital, Inje University, Busan, South Korea; 10 Department of Cardiovascular Medicine, St. Vincent’s Hospital, The Catholic University of Korea, Seoul, South Korea; 11 Department of Cardiovascular Medicine, Gil Medical Center, Gachon University, Incheon, South Korea; 12 Department of Cardiovascular Medicine, Gangnam Severance Hospital, Yonsei University, Seoul, South Korea; 13 The Heart Center, Chosun Medical Center, Gwangju, South Korea; 14 Department of Cardiovascular Medicine, Guro Hospital, Korea University, Seoul, South Korea; 15 Department of Cardiovascular Medicine, Samsung Medical Center, Sungkyunkwan University, Seoul, South Korea; 16 Division of Cardiology, Department of Internal Medicine, Seoul National University Bundang Hospital, Seongnam, South Korea; 17 Division of Cardiology, Seoul St. Mary’s Hospital, The Catholic University of Korea, Seoul, South Korea; Fiji national University School of Medicine, FIJI

## Abstract

**Background:**

The interaction between smoking and the use of antiplatelet agents on the prognosis of vasospastic angina (VA) is rarely investigated.

**Methods:**

VA-Korea is a nation-wide multi-center registry with prospective design (n = 1812). The primary endpoint was the composite occurrence of acute coronary syndrome (ACS), symptomatic arrhythmia, and cardiac death. Log-rank test and Cox proportional hazard model were for statistical analysis. Also, we conducted interaction analysis in both additive and multiplicative scales between smoking and antiplatelet agents among VA patients. For additive scale interaction, relative excess risk due to interaction (RERI) was calculated and for multiplicative scale interaction, the ratio of hazard ratio (HR) was calculated. All statistical analysis conducted by Stata Ver 16.1.

**Results:**

Patients who were smoking and using antiplatelet agents had the highest incidence rate in the primary composite outcome. The incidence rate was 3.49 per 1,000 person-month (95% CI: 2.30-5.30, log-rank test for primary outcome p = 0.017) and HR of smoking and using antiplatelet agents was 1.66 (95%CI: 0.98-2.81). The adjusted RERI of smoking and using antiplatelet agents was 1.10 (p = 0.009), and the adjusted ratio of HR of smoking and using antiplatelet agents was 3.32 (p = 0.019). The current study observed the interaction between smoking and using antiplatelet agents in both additive and multiplicative scales.

**Conclusions:**

Smoking was associated with higher rates of unfavorable clinical outcomes among VA patients taking antiplatelet agents. This suggested that VA patients, especially those using antiplatelet agents should quit smoking.

## Introduction

The prognosis of vasospastic angina (VA) is known to be better than acute coronary syndrome (ACS). Previous studies showed that angina at rest, ST-segment elevation during angina, coronary arterial stenosis were linked with worse prognosis among VA patients [1, 2]. Antiplatelet agents have been used for primary prevention of cardiovascular disease among high risk patients of coronary artery disease (CAD) like diabetes [3]. Antiplatelet agents are usually prescribed for secondary prevention for those with combined atherosclerotic CAD or receiving percutaneous coronary intervention (PCI) in VA patients. However, the use of antiplatelet agents, even low dose aspirin, showed controversial results on the prognosis of patients with VA [4–7]. Several studies argued that the use of aspirin might be linked to unfavorable prognosis, but others disputed that [4–7].

Smoking is the well-known risk factor for developing vasospasm [8], and can lead to poor clinical outcomes in VA patients [9–11], as well as an established risk for atherosclerotic CAD [12]. Smokers in VA patients who had risk factors or established CAD, particularly those with coronary stents, were likely to take antiplatelet agents. So the relationship between smoking and the use of antiplatelet agents in VA patients need to be sought. Smoking can influence platelet functions [13], and can inhibit the activity of antiplatelet agents [14, 15].

In this regard, the impact of smoking on the prognosis of VA patients who are taking antiplatelet agents should be investigated. However, as far as we know, no previous study has yet explored the interaction of antiplatelet agents and smoking on the prognosis in those with VA. Interaction analysis, both additive and multiplicative scales of interaction which theoretical epidemiologists recommend, could estimate this relationship of those in VA patients [16, 17]. Thus, this study aims to explore the interaction of smoking and antiplatelet agents on the prognosis of VA.

## Methods

### Participants

VA-Korea (Variant angina Korea) is a nation-wide prospective cohort based on the multi-center registry. The registry enrolled patients who had chest pain suggesting of VA and undertaking CAG and an ergonovine (EG) provocation test. Patients with aged 18 or over were candidates. Patients who had catheter-induced spasm during CAG, malignancy, end-stage renal disease, or inflammatory disease were excluded.

A total of 2960 patients were enrolled from May 2010 to June 2015 in 11 tertiary hospitals in Korea. One thousand eight hundred ninety-two patients (n = 1892) had positive results (680 definite and 1212 intermediate) in their provocation tests. Definite spasm is defined as total or subtotal (>90% luminal diameter narrowing) occlusion of coronary artery accompanied by ischemic symptoms and/or electrocardiographic (ECG) changes, and intermediate spams define as 50% to 90% luminal narrowing with or without ischemic symptoms and/or ECG changes during provocation test [18]. One thousand eight hundred thirty-eight (1838) patients followed the study and 54 patients were lost during the follow-up period. Information on both smoking status and use of antiplatelet agents were not gathered among 26 participants. In the final analysis, 1812 patients were included. Patients with positive EG provocation test results and spontaneous vasospasm received medical treatment, including calcium channel blockers and other vasodilators during follow-up. Patients were divided into four groups according to their differing use of smoking and antiplatelet agents at the enrollment of study: non-smoker and no-antiplatelet agents, smoking only, antiplatelet agents only, and both smoking and antiplatelet agents. Participants were followed up for three years and investigated during this time for clinical events.

Among the study participants, 820 patients were taking antiplatelet agents. Six hundred and thirty four (634) patients took aspirin, 56 patients were taking clopidogrel, and 130 patients took both aspirin and clopidogrel.

The study protocol was approved by the ethical review board of Hallym University Hospital (Hallym University Hospital IRB: No. 2010-I007) and each participating hospital. All procedures and methods were undertaken in accordance with the ethical guidelines of each hospital. Written informed consent was given to all participants of the study.

### Primary endpoints

The primary composite endpoints were death from cardiac causes, acute coronary syndrome (ACS), and new-onset symptomatic arrhythmia during the 3-year follow-up. ACS was defined as continuous or recurrent ischemic chest pain lasting more than 20 minutes with evidence of ischemic ECG changes and/or increase of cardiac markers, including myocardial infarction. Ischemic ECG changes were defined as followings: an ST elevation (0.1 mV or more), an ST depression (0.1 mV or more), a T-wave inversion, or a new occurrence of left bundle branch block (LBBB), which were recorded in at least two contiguous leads on the 12-lead ECG [19–21]. Symptomatic arrhythmia is defined as new-onset symptomatic premature beats, atrioventricular block, sick-sinus rhythm, atrial or ventricular tachycardia/fibrillation [22]. ECG was regularly checked during the outpatient follow-up and during emergency visits. When patients had symptoms related to arrhythmia, 24 hour Holter monitoring was undertaken. All adverse events of interest were confirmed through source document review, including medical records as well as telephone interviews were adjudicated by the local events committee.

Detailed procedures on coronary angiography, ergonovine provocation test and interpretation of ergonovine provocation test results were reported previously [7].

### Statistical analysis

For continuous variables, means and standard deviations were presented, and the differences of means between the groups were examined by the analysis of variance (ANOVA). For categorical variables, numbers and proportions were demonstrated, and chi-square tests were carried out. Each incidence rates and 95% confidence interval (CI)s were demonstrated according to both the use of antiplatelet agents and current smoking. Events per 1,000 person month were demonstrated for estimating the incidence rate of the primary endpoint (ACS, arrhythmia, or cardiac death). The log-rank test was conducted to compare the survival difference among 4 groups by the use of antiplatelet agents and smoking status. In addition, Cox proportional hazard regression was conducted to estimate the survival difference among groups using antiplatelet agents and smoking status. The HRs and the 95% CIs were presented. For the interaction analysis, post-estimation commands of Stata were utilized after conducting Cox proportional hazard regression. Linear combination command was used for ratios of HR, and nonlinear combination command was used for relative excess risk due to interaction (RERI). When p was less than 0.05, it was considered statistically significant. Statistical analysis was conducted by Stata ver. 16.1 (Stata Corp, College Station, Texas).

### Interaction analysis by additive scale and multiplicative scale

We followed the recommendation of theoretical epidemiologists. When the interaction analysis is conducted, the recommendation encourages both additive scale and multiplicative scale to provide sufficient information on interaction [16, 17].

RERI can be estimated by the following formula.

RERI = HR combination of smoking and antiplatelet agents – HR only smoking – HR only antiplatelet agents + 1

When RERI is larger than 0, It indicates that there is a supra-additive interaction.

Ratio of HR is estimated by the following formula.

Ratio of HR = HR combination of smoking and antiplatelet agents / (HR only smoking * HR only antiplatelet agents)

When ratio is larger than 1, It indicates that a combined effect is larger than the product of the two separate effects.

## Results

### Baseline demographic and clinical characteristics of study participants

Demographic and clinical characteristics were presented in Table 1. Total study population was 1812. The number of 4 groups are as follows: 742 were in non-smoking and no use of antiplatelet agents group, 250 in smoking and no use of antiplatelet agents group, 568 in non-smoking and use of antiplatelet agents group, and 252 in both smoking and using of antiplatelet agents group. Mean age ranged from 49.7 to 59 years (with differences in each group). Participants’ ages were higher among non-smokers. Sex, past medical history of hypertension, diabetes, coronary heart disease (CHD), percutaneous coronary intervention (PCI) history, spasm severity, and presence of atherosclerosis were statistically different among the groups by smoking and using antiplatelet agents. Percentages of male were higher among smokers. Medical history of diabetes mellitus, coronary heart disease (CHD), percutaneous coronary intervention (PCI) were highest among the group with both smoking and using antiplatelet agents. Percentage of definite spasm and atherosclerosis at angiography were the highest among the groups with smoking and using antiplatelet agents. Lipid profiles were statistically different among groups. In particular, high-density lipoprotein cholesterol (HDL-C) levels were lower, and triglyceride levels were higher in smokers. In general, unfavorable basal clinical conditions were observed among patients who were smoking and using antiplatelet agents.

**Table 1:** Basal demographic and clinical characteristics of study participants by smoking and use antiplatelet agent.

	Smoking (-) & AntiPLTs (-)		Smoking (+) & AntiPLTs (-)		Smoking (-) & AntiPLTs (+)		Smoking (+) & AntiPLTs (+)		
	n = 742		n = 250		n = 568		n = 252		
	Mean	sd	Mean	sd	Mean	sd	Mean	sd	p
Age	54.71	11.21	49.67	10.88	58.96	10.97	52.82	10.07	<0.001
BMI	28.78	91.44	24.81	3.74	30.04	105.94	24.55	3.06	0.765
SBP	126.69	44.15	126.03	19.60	125.82	18.92	129.53	19.05	0.471
DBP	76.74	11.68	78.32	12.24	75.55	12.63	79.71	12.61	<0.001
Total cholesterol	175.00	36.33	180.58	34.32	170.76	37.13	172.78	35.14	0.006
TG	131.55	93.98	175.88	119.78	130.27	83.97	176.84	145.37	<0.001
HDL-C	47.70	12.96	44.56	10.97	47.58	12.66	44.30	14.10	<0.001
LDL-C	104.48	30.85	106.90	31.80	102.71	32.22	99.87	30.72	0.107
hsCRP	0.73	4.24	1.68	11.42	0.79	7.80	0.53	1.49	0.313
CKMB	4.67	11.82	8.23	44.59	5.52	19.46	7.66	25.92	0.218
Troponin-I	0.32	2.92	0.30	1.59	1.00	6.77	0.63	2.97	0.232
LV-EF	64.69	6.68	64.44	5.97	64.23	6.72	64.21	7.14	0.637
	n	percent	n	percent	n	percent	n	percent	
Sex (male)	350	47.17	226	90.4	306	53.87	239	94.84	<0.001
HTN	241	32.48	70	28.00	276	48.59	99	39.44	<0.001
DM	58	7.82	20	8.03	61	10.76	33	13.15	0.046
Dyslipidemia	119	16.04	40	16.06	90	15.96	49	19.60	0.570
CHD	73	9.84	14	5.62	98	17.28	35	13.94	<0.001
PCI	6	0.81	1	0.40	22	3.87	10	3.97	<0.001
Definite spasm	218	29.38	103	41.20	215	37.85	128	50.79	<0.001
Atherosclerosis > 50%	25	3.37	10	4.00	63	11.09	35	13.89	<0.001

antiPLT, antiplatelet agents; sd, standard deviation; BMI, body mass index; SBP, systolic blood pressure; DBP, diastolic blood pressure; TG, Triglyceride; HDL-C, high-density lipoprotein cholesterol; LDL-C; low-density lipoprotein cholesterol; hs-CRP; high sensitivity C-reactive protein; CK-MB; creatine kinase-MB; LV-EF, left ventricular ejection fraction; HTN, hypertension; DM, diabetes mellitus; CHD, coronary heart disease; PCI, percutaneous coronary intervention.

### Incidence rate of primary composite outcome and its’ subcomponents

Primary endpoints were the composite of occurrence of cardiac death, acute coronary syndrome (ACS), and new-onset symptomatic arrhythmia during a 3-year follow-up. Incidence rates and unadjusted HRs for the composite primary endpoint and its’ subcomponents are presented in Table 2.

**Table 2:** Incidence rates and HRs (by uni-variable Cox-proportional hazard model) for primary outcome and sub-components.

	no	event (%)	Person-month	rate	95% CI		p*	HR	95% CI		p
Primary outcome							0.017				
Smoking(-) & AntiPLTs(-)	742	37	15497.59	2.39	1.73	3.30		ref			
Smoking(+) & AniPLTs(-)	250	7	5535.57	1.26	0.60	2.65		0.56	0.25	1.25	0.154
Smoking(-) & AniPLTs(+)	568	23	13940.17	1.65	1.10	2.48		0.77	0.46	1.30	0.332
Smoking(+) & AniPLTs(+)	252	22	6305.74	3.49	2.30	5.30		1.66	0.98	2.81	0.060
Arrhythmia							0.394				
Smoking(-) & AntiPLTs(-)	742	8	16240.16	0.49	0.25	0.99		ref			
Smoking(+) & AniPLTs(-)	250	3	5681.41	0.53	0.17	1.64		1.10	0.29	4.16	0.884
Smoking(-) & AniPLTs(+)	568	7	14432.23	0.49	0.23	1.02		1.01	0.37	2.79	0.980
Smoking(+) & AniPLTs(+)	252	7	6756.70	1.04	0.49	2.17		2.16	0.78	5.96	0.137
ACS							0.092				
Smoking(-) & AntiPLTs(-)	742	23	16017.41	1.44	0.95	2.16		ref			
Smoking(+) & AniPLTs(-)	250	5	5649.48	0.89	0.37	2.13		0.63	0.24	1.65	0.344
Smoking(-) & AniPLTs(+)	568	14	14380.81	0.97	0.58	1.64		0.68	0.35	1.33	0.264
Smoking(+) & AniPLTs(+)	252	15	6635.50	2.26	1.36	3.75		1.57	0.82	3.02	0.172
Cardiac death							0.205				
Smoking(-) & AntiPLTs(-)	742	7	16386.04	0.43	0.20	0.90		ref			
Smoking(+) & AniPLTs(-)	250	0	5733.26	0.00	.	.					
Smoking(-) & AniPLTs(+)	568	2	14570.84	0.14	0.03	0.55		0.33	0.07	1.57	0.162
Smoking(+) & AniPLTs(+)	252	3	6941.73	0.43	0.14	1.34		1.05	0.27	4.08	0.939

rate: event/1000 person-month, HR: hazard ratio; P*: p values of log-rank test; AntiPLTs: antiplatelet agents.

The incidence rate of the primary composite outcome was higher in patients who were smoking and using antiplatelet agents; 3.49 per 1,000 person-month (95% CI: 2.30-5.30, log-rank test for primary outcome p = 0.017) and the HR of smoking and using antiplatelet agents was 1.66 (95%CI: 0.98-2.81). The incidence rate of new-onset symptomatic arrhythmia showed a higher trend in the group with smoking and using the antiplatelet agents. It was 1.04 per 1,000 person-month (95%CI: 0.49-2.17, log-rank test for arrhythmia p = 0.394), and the HR was 2.16 (95% CI: 0.78-5.96). However, there was not a statistical difference. Similarly, the incidence rate of ACS showed the trend of being highest in the group smoking and using antiplatelet agents, with 2.26 per 1,000 person-month (95% CI:1.36–3.75, log-rank test for ACS p = 0.092) and HR was 1.57 (95% CI:0.82-3.02). When it comes to cardiac death, we could not find statistically significant differences in incidence rates and HRs among groups by smoking and using antiplatelet agents.

### Interaction analysis of smoking and use of antiplatelet agents

Table 3 showed the interaction between smoking and the use of antiplatelet agents on the primary endpoint.

**Table 3:** Interaction analysis of smoking and antiplatelet agents on primary composite outcome.

	Smoking(-)	Smoking(+)	HR for smoking(-) vs smoking(+) within strata of antiplatelet agents
	HR(95% CI):p	HR(95% CI):p	HR(95% CI):p
Antiplatelet agents(-)	reference	0.56(0.24-1.31):0.180	0.56(0.24-1.31):0.180
Antiplatelet agents(+)	0.76(0.44-1.31):0.325	1.42(0.78-2.57):0.246	1.86(1.00-3.49):0.051
HR for antiplatelet agents(-) vs antiplatelet agents(+) within strata of smoking	0.76(0.44-1.31):0.325	2.53(1.07-5.98):0.034	
Measure of interaction on additive scale: RERI	1.10(0.28-1.92):0.009		
Measure of interaction on multiplicative scale: ratio of HRs	3.32(1.21-9.06):0.019		

CI, confidence interval; RERI, relative excess risk due to Interaction; HR is adjusted for sex, age, BMI, history of coronary heart disease, hypertension, diabetes, dyslipidemia, severity of spasm, severity of atherosclerosis; Ratios of HRs were estimated using the linear combination command, RERI was estimated using the nonlinear combination command after cox proportional hazard regressions (linear combination and nonlinear combination are post-estimation commands of Stata).

**Fig 1:**
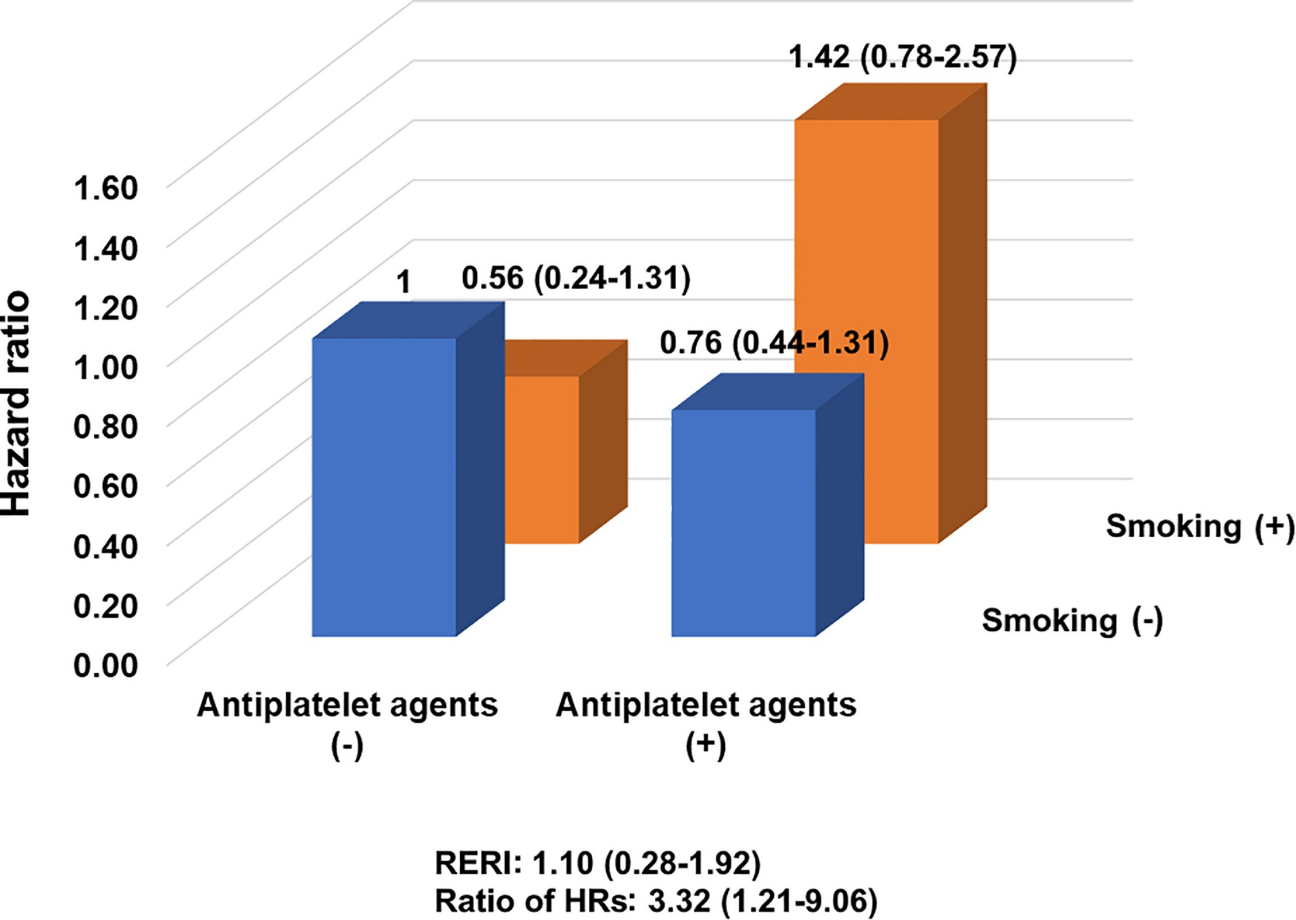
Interaction of smoking and antiplatelet agents on the primary composite outcome. RERI: relative risk of relative excess risk due to interaction, HR: hazard ratio.

Table 3 and Fig 1 showed the interaction of smoking and antiplatelet agents on the prognosis. In this model, age, sex, BMI, history of coronary heart disease, history of hypertension, history of diabetes, history of dyslipidemia, severity of atherosclerosis, and severity of spasm were included. Adjusted HR was 1.42 (95% CI: 078-2.57). RERI was 1.10 (95%CI:0.28-1.92) and the ratio of HR was 3.32 (95%CI:1.21-9.06). This study observed both a supra-additive interaction and a supra-multiplicative interaction between smoking and the using of antiplatelet agents on the primary outcome.

Table 4 demonstrated the result of interaction analysis when additional covariates were included in the model.

**Table 4:** Interaction analysis of antiplatelet agents and smoking on primary composite outcome additionally HDL cholesterol, TG, total cholesterol, and diastolic blood pressure were adjusted.

	Smoking(-)	Smoking(+)	HR for smoking(-) vs smoking(+) within strata of antiplatelet agents
	HR(95% CI):p	HR(95% CI):p	HR(95% CI):p
Antiplatelet agents(-)	reference	0.76(0.29-1.95):0564	0.76(0.29-1.95):0568
Antiplatelet agents(+)	1.13(0.61-2.11):0.691	1.77(0.89-3.53):0.104	1.56(0.79-3.08):0.199
HR for antiplatelet agents(-) vs Antiplatelet agents(+) within strata of smoking	1.13(0.61-2.11):0.691	2.34(0.92-5.92):0.074	
Measure of interaction on additive scale: RERI	0.88(-0.27-2.03):0.136		
Measure of interaction on multiplicative scale: ratio of HRs	2.06(0.70-6.37):0.198		

CI, confidence interval; RERI, relative excess risk due to Interaction; HR is adjusted variables for Table 3 sex, age, BMI, history of coronary heart disease, hypertension, diabetes, dyslipidemia, severity of spasm, severity of atherosclerosis, total cholesterol, TG, and HDL Cholesterol; HRs and ratios of HRs were estimated using the linear combination command, RERI was estimated using the nonlinear combination command after Cox proportional hazard regression (linear combination and nonlinear combination are post-estimation commands of Stata).

Total cholesterol, triglyceride, and HDL-cholesterol were additionally included in the model. In this model, RERI was 0.88 (95%CI:-0.27-2.03; p = 0.136) and the ratio of HR was 2.06 (95%CI: 0.70-6.37; p = 0.198). RERI was larger than 0 and the ratio of HR was larger than 1. However, the amplitudes of both RERI and the HR ratio decreased, and we did not observe statistical significance.

## Discussion

To the best knowledge, the current study may be the first study to investigate the interaction between smoking and antiplatelet agents on the prognosis of vasospastic angina following the recommendation for interaction analysis by theoretical epidemiologists. The epidemiologists recommended that researchers should measure in both additive scale and multiplicative scale in order to provide sufficient information to readers [16, 17]. In this study, both supra-additive and supra-multiplicative interactions were observed between smoking and using antiplatelet agents. RERI was 1.10 (95%CI:0.28-1.92), and the ratio of HR was 3.32 (95%CI:1.21-9.06). It implies that smoking can aggravate the prognosis of VA patients who were taking antiplatelet agents and the interaction between smoking and antiplatelet agents may contribute this unfavorable prognosis. Smoking is one of the most important modifiable risk factors of CAD. The harmful effects of smoking can be attributed to many pathophysiologic changes including, hemodynamic change of systemic vasculature and coronary artery, inflammation, oxidative stress, endothelial dysfunction, unfavorable changes in lipid metabolism, hypercoagulative state, thrombosis, and platelet aggregation [23–27]. Smoking also can induce coronary vasospasm and worsens the prognosis of VA [11]. It has been yet controversial that the use of antiplatelet agents can affect the prognosis among VA patients. Some studies reported worse prognoses of VA in using low dose aspirin [4, 6]. In contrast, other studies observed that aspirin was neutral [5, 7]. A study from Korea reported the interaction between using dual antiplatelet agents and smoking on prognosis in the subgroup analysis [7]. It is unclear whether a worse prognosis is related to the combined effect of antiplatelet agents and smoking per se or unfavorable baseline conditions of patients who are using the antiplatelet agents. The result of the current study suggests that cessation of smoking should be emphasized among VA patients who are taking antiplatelet agents for a better prognosis.

Although we found the interaction of smoking and antiplatelet agents and its impact on the prognosis of VA, this study could not investigate the more detailed mechanisms of the interaction. We can presume that smoking might alter the hemodynamics of blood vessels, endothelial function, platelet aggregation, thrombosis, and blood coagulation [24–26, 28–30], and this resulted in the inference on functions of antiplatelet agents directly or indirectly [14, 15]. Although no study examined the direct interaction between smoking and aspirin, some demonstrated the clue in that aspirin could not overcome the more potent effect of smoking on platelet aggregation and thrombosis [13, 31]. It implies the interaction might be present and smoking could lead to platelet aggregation and thrombosis.

Another possible mechanism of interaction is general metabolic changes induced by smoking particularly alteration of lipid metabolism [24]. Smokers have higher blood triglyceride levels and a lower blood HDL-cholesterol levels [27]. As seen in the Table 4, when total cholesterol, triglyceride, HDL-Cholesterol were additionally included in the model, the statistical significance of interactions was not seen in either additive scale or multiplicative scale. RERI was 0.88 (95%CI:-0.27-2.03, P = 0.136) and ratio of HR was 2.06 (95%CI: 0.70-6.37, P = 0.198). Thus, triglyceride and HDL-cholesterol might be contributors of interaction between smoking and using of antiplatelet agents.

The observed interaction in the current study may be ascribed to unfavorable basal characteristics of patients who were using antiplatelet agents. Usually, antiplatelet agents are prescribed for primary and secondary prevention. As seen in Table 1, patients using antiplatelet agents have higher prevalences of hypertension, diabetes, and CAD. Smoking might interact with unfavorable basal conditions of antiplatelet users, and smoking could aggravate patients’ general conditions using the antiplatelet agents. Finally, we could not exclude the possibility that residual confounders may affect the result. Although this study utilized multiple regression models to adjust potential confounders, observational studies cannot fully control residual confounders by using the statistical models.

### Strengths and limitations

The main strength of study has a larger number of study participants with multi-center (n = 1812). The prospective cohort design is another strength of the study. Three aspects of study limitations should be mentioned. The main limitation is that this study is an observational study. Observed results could not establish the causality due to residual confounders. In particular, patients who were taking antiplatelet agents have unfavorable basal clinical conditions. They had higher prevalences of hypertension, diabetes, and CAD. These unfavorable basal conditions of VA patients taking antiplatelet agents may contribute the worse outcomes. Secondly, this study could not explore more detailed mechanisms of the interaction between smoking and antiplatelet agents. Thirdly, this study could not consider changes in smoking and antiplatelet agent use because we gather the information on smoking and using of antiplatelet agents at the point of enrollment. We hope that future studies investigate how smoking changes metabolisms related to the interaction between smoking and the use of antiplatelet agents, such as changes in platelet functions, thrombosis, functions of endothelium, and lipid metabolisms.

## Conclusions

We observed the interaction of smoking and antiplatelet agents on the prognosis of patients with VA. The result of the current study indicated that smoking could lead to a worse prognosis of VA, particularly among those who were using antiplatelet agents. Interactions were observed in both additive and multiplicative scales. Quitting smoking could not be overemphasized among Va patients for a better prognosis.
